# S6-4 How to engage community-dwelling older adults in fall prevention programs? – a qualitative study into potential strategies

**DOI:** 10.1093/eurpub/ckad133.031

**Published:** 2023-09-11

**Authors:** Meike van Scherpenseel, Lidia van Veenendaal, Saskia te Velde, Amber Ronteltap

**Affiliations:** HU University of Applied Sciences Utrecht; HU University of Applied Sciences Utrecht; HU University of Applied Sciences Utrecht; HU University of Applied Sciences Utrecht

## Abstract

**Aim:**

Fall rates and fall-related injuries among community-dwelling older adults (≥65 years) will likely increase due to the rapidly aging population worldwide. Therefore, fall prevention has become an international priority. Fall prevention programs (FPPs), consisting of strength and balance exercises, have been proven effective in reducing the number of falls among older adults. However, in practice, professionals find it difficult to enroll participants for FPPs. Therefore, this study aims to identify effective strategies that promote engagement in FPPs among older adults.

**Methods:**

Based on previously identified barriers and facilitators for participation in FPPs by older adults, six strategies were designed using Intervention Mapping: 1) reframing; 2) informing about benefits; 3) raising awareness of risks; 4) involving social environment; 5) offering tailored intervention; 6) arranging practicalities. Strategies were presented and validated during semi-structured interviews with older adults (n = 12) at risk of falling. Interviews were recorded, transcribed and analyzed following a hybrid qualitative thematic method.

**Results:**

Analysis showed that reframing 'aging’ and 'fall prevention' is important. Within this strategy, themes emerged related to respondents’ preference to be approached differently, taking a ‘life course’ perspective about falls, and avoiding confronting terms. Furthermore, the strategy 'informing about benefits' (e.g. ‘living independently for longer') could contribute to understanding the relevance of participating in FPPs. Other strategies were considered important to take into account too, but were less emphasized.

**Conclusion:**

This study provides insight into possible strategies to stimulate older adults to participate in FPPs. Reframing ‘aging’ and 'fall prevention' may facilitate the conversation about fall prevention, which can be achieved by communicating differently about the topic, for example ‘staying fit and healthy’, while focusing on the benefits of participating in an FPP. Developing practical recommendations about how to apply the strategies, is an area for future research, as this could help health and social care professionals to enhance older adults’ participation in FPPs. Currently, the strategies are further developed to be applied and evaluated for effectiveness in multiple field labs in a central Dutch region (Utrecht).

**Funding source:**

This research was co-funded by Regieorgaan SIA, part of the Netherlands Organization for Scientific Research (NWO).

